# Immunomodulatory effects of *Eimeria maxima* surface antigen (EmSAG) as an IFN-γ inhibitory molecule on peripheral blood mononuclear cells (PBMCs) and T cell subsets in chickens

**DOI:** 10.1186/s13567-025-01535-7

**Published:** 2025-05-19

**Authors:** Xianglin Pu, Yiyuan Zhang, Xinmei Huang, Mingmin Lu, Lixin Xu, Ruofeng Yan, Xiangrui Li, Xiaokai Song

**Affiliations:** 1 Ministry of Education (MOE) Joint International Research Laboratory of Animal Health and Food Safety, College of Veterinary Medicine, Nanjing Agricultural University, Nanjing, 210095 China; 2 Institute of Veterinary Medicine, Jiangsu Academy of Agricultural Science, Nanjing, 210014 China

**Keywords:** IFN-γ inhibitory molecule, EmSAG, T cell subsets, immune evasion, immunomodulation

## Abstract

*Eimeria maxima* (*E. maxima*) infection inhibits the expression of IFN-γ, a cytokine that is essential for the Th1 immune response and plays a key role in combating this parasite. In our preliminary investigations, we identified the *E. maxima* surface antigen (EmSAG) as an inhibitory molecule of IFN-γ. EmSAG was screened and characterised from an *E. maxima* sporozoite cDNA expression library. The present study aimed to evaluate the immunomodulatory effects of EmSAG on chicken peripheral blood mononuclear cells (PBMCs) and various T cell subsets. We analysed cell proliferation, nitric oxide (NO) release, and cytokine transcription. The results revealed that EmSAG boosts PBMC proliferation and promotes differentiation of CD4^+^/CD8^+^ T cells. Additionally, stimulation with EmSAG significantly inhibited NO release and IFN-γ transcription while enhancing the transcription of IL-4, IL-10, and TGF-β1 in chicken PBMCs. The sorting purity of T cell subsets was as follows: CD8^+^ (96.90%), CD4^+^ (86.25%), CD4^+^CD25^−^ (89.14%), and CD4^+^CD25^+^ regulatory T cells (Tregs; 92.16%). These purified subsets were co-incubated with EmSAG to analyse the transcription of hallmark cytokines associated with Th1, Th2, and Treg responses. EmSAG significantly inhibited the transcription of IFN-γ and IL-2 in both CD4^+^ and CD8^+^ T cells, while promoting the expression of IL-10, TGF-β1, and CTLA-4 in Tregs. Moreover, depletion of CD25^+^ cells reversed the EmSAG-induced suppression of IL-2 transcription and reduced its stimulating effects on IL-4 and IL-10 transcription in CD4^+^CD25^−^ T cells. These findings highlight the role of EmSAG as an inhibitor of IFN-γ, facilitating immune evasion by attenuating the Th1 immune response and modulating Treg cell function. This study provides critical insights into the immune evasion mechanisms utilised by chicken coccidia.

**Supplementary Information:**

The online version contains supplementary material available at 10.1186/s13567-025-01535-7.

## Introduction

Chicken coccidiosis is a parasitic enteric disease caused by single or mixed infections of various *Eimeria* species. It continues to impose significant economic burdens on the global poultry industry, leading to serious clinical symptoms such as mortality, bloody feces, weight loss, and reduced egg production [1]. A particular concern is the disease’s ability to make chickens more susceptible to secondary infections by opportunistic pathogens such as *Clostridium perfringens* [2, 3].

The rise of widespread drug resistance in *Eimeria* strains, along with increasing regulatory restrictions on anticoccidial drug residues in poultry products, has significantly hindered current control strategies [4, 5]. Although traditional live attenuated vaccines offer some protection, their practical application is fraught with challenges, such as the risk of parasite spread, high production costs, and manufacturing limitations [1, 4].

Therefore, gaining a deeper understanding of the competition between chicken coccidia and the host immune system, as well as the possible mechanisms of immune evasion, is essential for advancing the study of parasite pathogenicity and developing novel vaccines.

In the long-term evolution process of parasites, they have developed sophisticated strategies to evade the host's immune system. This is achieved by secreting immunomodulatory factors and manipulating immune responses, which enables them to survive, reproduce, and induce diseases. Parasite-derived enzymes, or surface glycoproteins, exert various immunosuppressive effects through antioxidant activity, inflammatory suppression, immune cell apoptosis induction, and inhibition of lymphocyte activation.

Protozoan infections, for example, can inhibit the expression of Th1 cytokines in the host, and various cytokine inhibitory molecules have been identified [6–9]. For instance, the *Trypanosoma cruzi* (*T. cruzi*) membrane glycoprotein AGC10 (glycosylphosphatidylinositol-anchored) impairs macrophage functions and suppresses the production of IFN-γ/IL-2 in PBMCs [10]. Similarly, the lipophosphoglycan (LPG) from *Leishmania shawi* (*L. shawi*) modulates the cytokine profiles of peritoneal macrophage by reducing IL-12 and elevating IL-10 [11].

These coordinated mechanisms collectively create an immunosuppressive microenvironment that supports parasitic survival and chronic infection. In the context of chicken coccidia infections, Cornelissen et al. reported that, at 4 days post-infection (dpi) with *E. maxima*, *acervulina* and *tenella,* there was a suppression of IFN-γ mRNA across different intestinal segments (jejunum/duodenum/cecum), along with increased expression of IL-4/IL-10 [12]. Additionally, Walston's study demonstrated a significant reduction in IFN-γ mRNA levels in the spleen by 11 dpi, which decreased to half the level observed in uninfected controls [13].

These studies suggest that other molecules may regulate or inhibit IFN-γ and related cytokines during chicken coccidia infections, although the molecular mediators involved remain largely uncharacterised. Notably, Chen et al. identified *E. maxima* surface antigen (EmSAG) as an IFN-γ inhibitory molecule. They demonstrated that EmSAG could inhibit IL-12p40 secretion in chicken HD11 macrophages and bone marrow-derived dendritic cells (BMDCs). Mechanistic studies showed that EmSAG activates phosphorylation of extracellular signal-regulated kinase 1/2 (ERK 1/2) within the mitogen-activated protein kinase (MAPK) cascade, establishing a regulatory axis for IL-12 suppression [14].

It is suggested that molecules secreted by *Eimeria* parasites may suppress the host's IFN-γ response by inhibiting its expression, ultimately enhancing the survival of the parasites. The limited identification of *Eimeria’s* IFN-γ inhibitory molecules highlights the urgent need to characterise such molecules. Doing so could reveal new targets for disrupting *Eimeria’s* immune evasion and inform the development of next-generation control strategies.

Pathogens evade host immunity by suppressing essential immune cell functions, which include activation, proliferation, differentiation, and regulatory processes. CD4^+^CD25^+^ regulatory T cells (Tregs) play a crucial role as mediators through contact-dependent mechanisms and the production of immunosuppressive cytokines.

Parasite-activated Tregs orchestrate immune suppression by secreting regulatory cytokines (IL-10, TGF-β, and IL-35). They also release cytotoxic mediators (granzyme B and perforin-1), and upregulate cytotoxic T lymphocyte-associated protein 4 (CTLA-4) [15–17]. These effectors can inhibit Th1/Th2 polarisation, CTLs cytotoxicity, and NO production, all of which contribute to parasite survival and prevent excessive immunopathological reactions.

Additionally, avian coccidia trigger a significant increase in the proportion of Tregs in chickens, suggesting that *Eimeria* induces the activation of regulatory T cell subsets [18]. An oral challenge with mixed *Eimeria* spp. (*E. tenella*, *E. acervulina*, and *E. maxima*) reduces the population of CD4^+^ T cells in the cecal mucosa while increasing the proportion of Tregs and their secreted specific cytokines (IL-10 and TGF-β) [13, 19]. These findings indicate that chicken Tregs and the immunosuppressive molecules they produce may play a role in the pathogenesis of chicken coccidiosis.

A potential mechanism for immune evasion by *Eimeria* spp. involves their induction of Treg activation in the host, leading to high expression of immune inhibitory cytokines (IL-10 and TGF-β) and a subsequent suppression of IFN-γ-mediated Th1 responses. This process promotes parasite survival and infection [20].

Building on previous evidence that EmSAG, an *E. maxima*-derived IFN-γ inhibitor, suppresses IL-12 in innate immune cells [14], this study explores its role in T cell-mediated adaptive immunity. We first assessed the functionality of PBMCs by measuring cell proliferation, CD4^+^/CD8^+^ T cells proportion, NO release, and cytokines transcription. We then analysed the specificity of cytokine responses induced by rEmSAG stimulation in chicken CD4^+^, CD8^+^, and CD4^+^CD25^+^ T cells. Finally, we conducted a CD25^+^ cell depletion assay to examine the dynamic changes in Th1/Th2/Treg cytokines, thereby elucidating the role of Tregs in modulating CD4^+^ T cell functionality. This study not only provides critical insights into the immune evasion strategies of *Eimeria* spp., but also establishes a theoretical foundation for improving the effectiveness of coccidiosis vaccines in chickens.

## Materials and methods

### Animals, strain and plasmid

In this study, newborn Hy-Line white chicks used for experiments and were raised in a thoroughly cleaned feeding room. The floors, walls, and cages were sanitized using hot water at temperatures exceeding 90 °C. All feed troughs and utensils in the room were soaked in hot water above 90 °C for more than 10 min to inactivate any potential remaining coccidia oocysts. Additionally, the room and all utensils were routinely disinfected with a 0.1% benzalkonium bromide solution to eliminate any potential contamination from other pathogens. The chicks had unrestricted access to drinking water and feed, which did not contain anticoccidial drugs.

Sporulated oocysts of the *E. maxima* Jiangsu strain were stored in a 2.5% (w/v) potassium dichromate solution at 4 ℃. *Eimeria*-free chicks were orally infected every three months for rejuvenation and propagation purposes. The pET-32a-EmSAG plasmid, which encodes the EmSAG antigen (RefSeq ID: XP_013337465.1), was constructed and maintained in our laboratory.

### Purification of rEmSAG

*E. coli* BL21 (DE3) containing the pET-32a-EmSAG plasmid was cultured in LB medium supplemented with ampicillin (100 µg/mL) at 37 ℃ with shaking at 180 r/min until the OD_600_ value reached approximately 0.6. The recombinant protein EmSAG (rEmSAG) was expressed by adding Isopropyl β-D-thiogalactoside (IPTG; 100 mM; Yfxbio Biotech. Co., Ltd., Nanjing, China) and shaking the culture for 5 h under the same conditions. The bacterial cells were collected and then disrupted ultrasonically.

Subsequently, rEmSAG was purified using a His-tag purification column (Cytiva, Marlborough, MA, USA), and purity verification was performed using 12% (w/v) sodium dodecyl sulfate–polyacrylamide gel electrophoresis (SDS-PAGE). To eliminate endotoxin from the purified protein, a ToxinEraser^™^ Endotoxin Removal Kit (GenScript, Nanjing, China) was utilised. Finally, the protein concentration was determined using a Pierce^™^ BCA Protein Assay Kit (ThermoFisher Scientific, Waltham, MA, USA).

### Immunoblot analysis of rEmSAG

Sera were harvested from *E. maxima*-experimentally infected chickens as well as coccidia-free chickens using a previously established method [21]. The recognition of rEmSAG was evaluated through immunoblot analysis, utilising serum from *E. maxima*-infected chickens and a mouse-derived anti-His tag monoclonal antibody (Proteintech, Wuhan, China).

In brief, rEmSAG was separated using 12% SDS-PAGE and transferred to a polyvinylidene difluoride (PVDF) membrane (Merck, Darmstadt, Hessen, Germany) using a Trans-Blot^®^ Turbo^™^ Transfer System (Bio-Rad, Hercules, CA, USA). After the transfer, the PVDF membrane was blocked with 5% (w/v) skimmed milk (Solarbio, Beijing, China) for 2 h at room temperature. The membrane was then incubated overnight at 4 °C on a rotary shaker (80 r/min) with either *E. maxima*-infected chicken serum (1:100 dilution) or mouse-derived anti-His tag monoclonal antibody (1:8000 dilution). Coccidia-free chicken serum served as a negative control.

Following the antibody incubation, the membrane was rinsed six times with TBST (5 min per rinse) and then incubated with horseradish peroxidase (HRP)-conjugated goat anti-chicken IgY (H + L) (Abbkine, Atlanta, GA, USA) (1:5000 dilution) or anti-mouse IgG (H + L) (Affinity Biosciences, Cincinnati, OH, USA) (1:10 000 dilution) at 37 °C for 1 h. After the secondary antibody incubation, the membrane received additional TBST rinses and was reacted with Chemistar^™^ High-sig ECL Western Blotting Substrate (Tannon, Shanghai, China). The protein bands were visualised using a Tannon 5200 series automatic chemiluminescence imaging system (Tannon, Shanghai, China).

### Separation and culture of chicken PBMCs

Chicken PBMCs were isolated using a previously described density gradient centrifugation protocol [22]. The freshly isolated PBMCs were washed with sterile phosphate-buffered saline (PBS) and then resuspended in RPMI 1640 complete culture medium (Gibco, Waltham, MA, USA). This medium contained 10% (v/v) fetal bovine serum (FBS; Excell Bio, Shanghai, China) and 1% (v/v) penicillin–streptomycin solution (Gibco, Waltham, MA, USA). The cell concentration was counted and adjusted to 1 × 10^6^ cells per 1 mL in the complete culture medium.

### Effect of rEmSAG on the proliferation of chicken PBMCs

To assess the proliferation effect of rEmSAG on chicken PBMCs, we used the CellTrace^™^ Cell Proliferation Kit (Invitrogen, Waltham, MA, USA) following the manufacturer’s instructions. First, a suspension of PBMCs was prepared based on experimental requirements and labelled with 1 μM CellTrace^™^ Far Red dye (Invitrogen, Waltham, MA, USA) working solution. The cell suspension was incubated with the dye at 37 °C with 5% CO_2_ for 20 min. Staining was then terminated by washing the cells with PBS (5 times suspension volume), followed by centrifugation and a brief incubation with complete medium at 4 °C for 5 min.

Next, the PBMCs were resuspended in freshly pre-warmed culture medium (37 °C) and seeded into 12-well cell culture plates. The cells were incubated for 48 h at 37 °C with 5% CO_2_ in the presence of the following treatments: rEmSAG at final concentrations of 10, 20, 40, and 80 μg/mL, PBS (negative control), pET-32a tag protein, and lipopolysaccharides (LPS; 2 μg/mL; positive control; Sigma-Aldrich, Saint Louis, MO, USA).

After incubation, the cells were transferred to 1.5 mL tubes, centrifuged at 300 × *g* for 10 min at 4 °C, rinsed twice with 1 mL PBS, and resuspended in 400 μL PBS. Additionally, two control groups were established: one group was stained but not cultured to determine the fluorescence intensity of the parent generation, while the other was cultured but not stained to determine the auto-fluorescence intensity of the progeny generation. Finally, the fluorescence intensity of all cells was analysed by flow cytometry (Beckman Coulter, Brea, CA, USA) to assess the cell proliferation index.

### Impacts of rEmSAG on the proportion of CD4^+^/CD8^+^ T lymphocytes in chicken PBMCs

PBMCs suspension was seeded into a 12-well cell culture plate and treated with varying final concentrations of rEmSAG (10, 20, 40, and 80 μg/mL) in a cell incubator for 24 h at 37 °C. Control groups included PBS, His-tagged pET-32a protein, and 2 μg/mL LPS.

After treatment, the cells were centrifuged at 300 × *g* for 10 min at 4 °C and resuspended in 100 μL PBS. Surface staining was performed by adding 1 μL of mouse-derived anti-chicken CD3, CD4 and CD8 antibodies (Southernbiotech, Birmingham, AL, USA) conjugated with fluorescein isothiocyanate (FITC), allophycocyanin (APC), and P-phycoerythrin (PE). The cells were incubated with the antibodies in the dark at 4 °C for 30 min.

Following the incubation, the cells were rinsed with 900 μL of PBS and then resuspended in 400 μL PBS. Multiple control groups need to be established for the subsequent flow cytometry serial gating strategy, including a blank control without any fluorescent staining, single-staining controls with different fluorescent antibodies, and fluorescence minus one (FMO) control. Finally, to determine the proportion of CD4^+^/CD8^+^ T lymphocytes, all cells were analysed using flow cytometry.

### Effects of rEmSAG on total nitric oxide release and cytokines transcription in chicken PBMCs

Fresh chicken PBMCs were resuspended in DMEM complete medium (Gibco, Waltham, MA, USA) containing 1% penicillin–streptomycin solution and 10% FBS, adjusting the cell density to 1 × 10^6^ cells per 1 mL. The chicken PBMCs were then stimulated with rEmSAG at concentrations of 10, 20, 40, and 80 μg/mL (*n* = 3 independent replicates per group) in a 12-well cell culture plate. Control groups included PBS, pET-32a tag protein, and 2 ug/mL LPS.

After incubation, the cell culture was centrifuged at 300 × *g* for 10 min at 4 °C to separate the supernatant and pellet for the determination of NO release and cytokine transcription levels. The concentrations of NO_2_^−^ and NO_3_^−^, which are stable metabolites of NO, in the supernatant were measured using a total nitric oxide (NO) assay kit (Beyotime, Nanjing, China) following the nitrite reductase method. The absorbance value at OD_540_ wavelength was recorded using a microplate reader (ThermoFisher Scientific, Waltham, MA, USA), and the total NO release levels for the different treatment groups were calculated by preparing a standard curve with sodium nitrite solution standards at concentrations of 0, 1, 2, 5, 10, 20, 40, 60, and 80 μM.

The cell pellet was transferred to 1.5 mL RNase-free tubes, and total RNA was extracted and converted into cDNA using the Total RNA Extraction Reagent (Vazyme, Nanjing, China) and HiScript III RT SuperMix (Vazyme, Nanjing, China), respectively. In brief, 1 μg of total RNA was reverse transcribed into 20 μL of cDNA, which was then diluted 5 times with RNase-free water (Transgen, Beijing, China).

Following our established protocol [22], a Quantitative PCR (qPCR) reaction system was prepared with a total volume of 10 μL using cytokine-specific primers detailed and validated in Table 1. The reaction mixture contained 5 μL of 2 × PerfectStartTM Green qPCR Super Mix (+ Dye II) (Transgen, Beijing, China), 0.2 μL of forward and reverse primers at a concentration of 10 μM each, 1 μL of diluted cDNA, and 3.6 μL of RNase-free water.

**Table 1:** **Primer sequences used for quantitative real-time PCR**

RNA target	Primer sequence (5' → 3')	Accession NO	Length of product
β-actin^a^	GCCAACAGAGAGAAGATGACAC	NM_205518	140
	GTAACACCATCACCAGAGTCCA		
IFN-γ^a^	ATCATACTGAGCCAGATTGTTTCG	Y07922	140
	TCTTTCACCTTCTTCACGCCAT		
IL-2^a^	TTCATCTCGAGCTCTACACACCAA	NM_204153	108
	TGTCATCTTCAGTTTCTTTCTTCAGAGT		
TNF-α^b^	AGTTCAGATGAGTTGCCCTTCCTG	XM_015294124	153
	TTCAGAGCATCAACGCAAAAGGGA		
IL-4^a^	AGCACTGCCACAAGAACCTG	NM_001007079	100
	CCTGCTGCCGTGGGACAT		
IL-10^a^	CTTTGGCTGCCAGTCTGTGTC	NM_001004414	94
	GCTCTGCTGATGACTGGTGCT		
TGF-β1^a^	GCCGACACGCAGTACACCAA	M31160	169
	TGCAGGCACGGACCACCAT		
CTLA-4^a^	CAAGATGGAGCGGATGTACC	NM_001040091	164
	TGGCTGAGATGATGATGCTG		

^a^Primers developed by Pu et al. [22]

^b^Primer described in this study

The qPCR reaction program was set up for a two-step qPCR assay. The initial step involved pre-denaturation at 94 °C for 30 s, followed by 40 cycle reactions of 94 ℃ for 5 s, and 60 ℃ for 30 s. The final melting curve consisted of 95 °C for 15 s, 60 °C for 60 s, and 95 °C for 15 s. The experimental data were analysed using the 2^−ΔΔCt^ method [23] to assess the effect of rEmSAG on cytokine transcription in chicken PBMCs.

### Immunomagnetic bead sorting of chicken T cell subsets

CD8^+^ T cells, CD4^+^CD25^−^ T cells, and CD4^+^CD25^+^ Tregs were sorted from freshly isolated chicken PBMCs following our established protocol [22]. The PBMCs were first incubated with mouse-derived anti-chicken CD8α or CD4 antibodies labelled with PE, along with anti-PE antibodies labelled with MicroBeads (Miltenyi Biotec, Bergisch Gladbach, North Rhine-Westphalia, Germany) for 30 min at 4 °C. The labelled cells were then transferred to a magnetic bead sorting system (Miltenyi Biotec, Bergisch Gladbach, North Rhine-Westphalia, Germany) for the sorting of chicken CD8^+^ or CD4^+^ T cells.

In a similar manner, chicken PBMCs were incubated with FITC-conjugated human anti-chicken CD25 antibodies (Bio-Rad, Hercules, CA, USA) and anti-FITC MultiSort MicroBeads antibodies (Miltenyi Biotec, Bergisch Gladbach, North Rhine-Westphalia, Germany) for 30 min at 4 °C. This step allowed for the separation of CD25^+^ cells and CD25^−^ PBMCs through a magnetic bead sorting system.

Subsequently, the sorted CD25^+^ cells or CD25^−^ PBMCs were further incubated with PE-labelled mouse anti-chicken CD4 antibodies and anti-PE MicroBeads to isolate CD4^+^CD25^+^ or CD4^+^CD25^−^ T cells. Finally, the purity of the various T cell subsets obtained through immunomagnetic bead sorting was confirmed using flow cytometry.

### Effects of rEmSAG on cytokines transcription in chicken T cell subsets

Freshly sorted chicken T cell subsets, including CD8^+^, CD4^+^, and CD4^+^CD25^+^ T cells were stimulated with 20 μg/mL rEmSAG at 37 °C for 6 h in 12-well cell culture plates. The control groups included PBS, pET-32a tagged protein, and 2 µg/mL LPS. After the incubation period, the cells were transferred to 1.5 mL tubes by centrifugation at 300 × *g* at 4 °C for 10 min. Total RNA was extracted from the cell pellet and reverse transcribed into cDNA. Subsequently, a qPCR assay was performed using the cDNA and cytokine-specific primers, detailed and validated in Table 1. Finally, the effects of rEmSAG on the transcription of cytokines associated with Th1, Th2, and Treg responses in different T cell subsets of chickens were assessed using the 2^−ΔΔCt^ method.

### Effects of rEmSAG on cytokines transcription of chicken CD4^+^CD25^−^ T cells following CD25^+^ cell depletion

CD4^+^CD25^−^ T cells were isolated by depleting CD25^+^ cells from chicken CD4^+^ T cells. The isolated cells were incubated with 20 μg/mL of rEmSAG at 37 °C with 5% CO_2_ for 6 h. Control groups included PBS, His-tagged pET-32a protein, and 2 µg/mL LPS in a 12-well cell culture plate. After incubation, total RNA was extracted from the cells and reverse transcribed into cDNA for qPCR experiments. CD4^+^ T cells were used as a reference to assess the potential regulatory effects of CD25^+^ cells by measuring the transcription levels of Th1, Th2, and Treg cytokines.

### Statistical analysis of data

The experimental data was analysed using SPSS 23.0 system software (IBM, Armonk, NY, USA). To assess the differences between treatment groups, we applied Duncan’s test within a one-way ANOVA framework. The results are presented as the mean ± standard deviation (SD). Different letters indicate a significant difference (*p* < 0.05), while the same letters denote no significant difference (*p* > 0.05). Furthermore, we compared the transcription of cytokines in CD4^+^ T cells before and after the depletion of CD25^+^ cells using an independent sample t-test. The levels of significance were defined as follows: *p < 0.05, **p < 0.01, and “ns” for nonsignificant results.

## Results

### Purification and immunoblot analysis of rEmSAG

**Figure 1:**
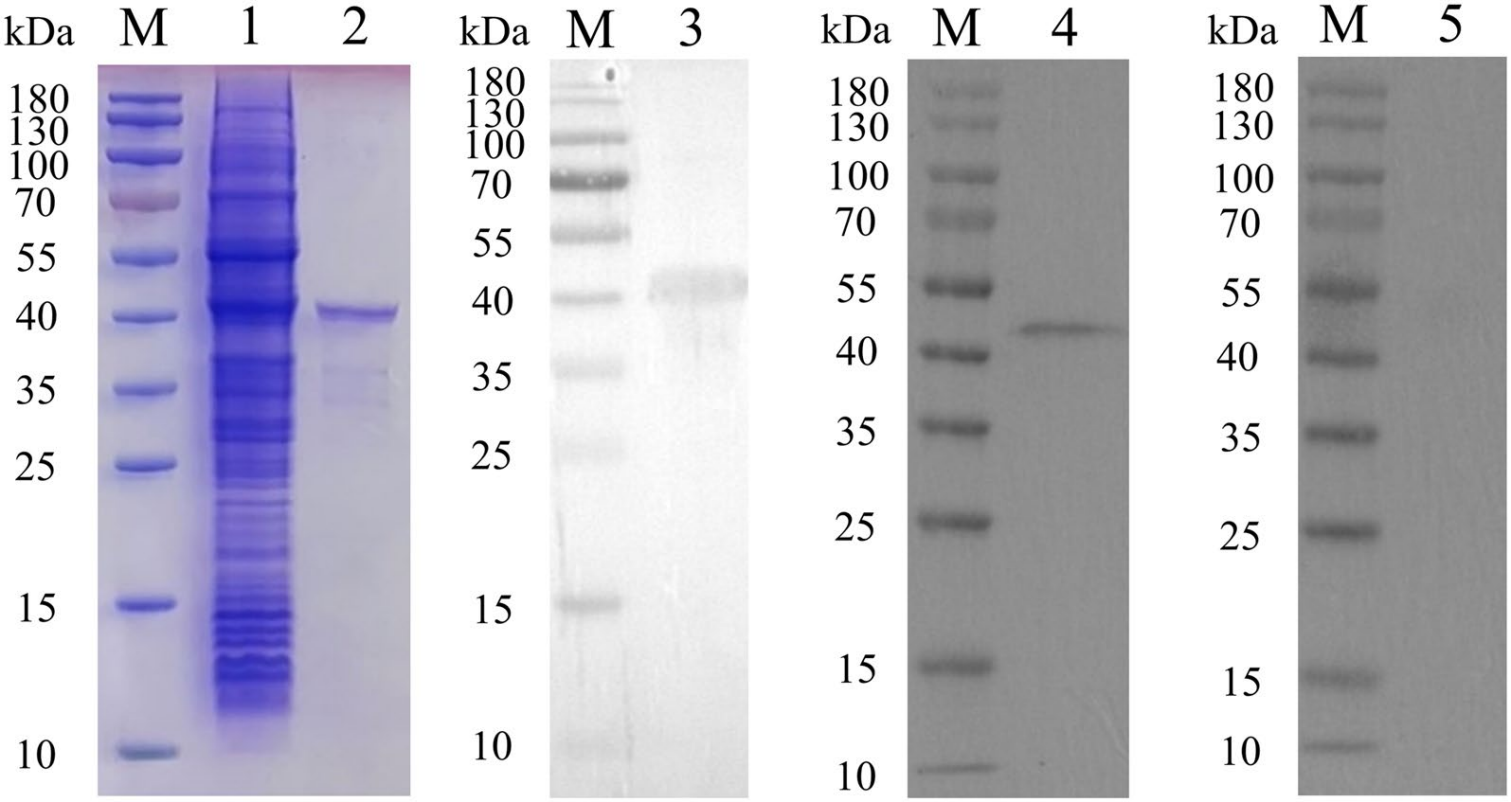
**Purification and immunoblot analysis of rEmSAG.** lane M: standard molecular marker for protein; lane 1: rEmSAG before purification; lane 2: purified rEmSAG; lane 3: His-tag in purified rEmSAG was identified by mouse-derived anti-His tag antibody; lane 4: purified rEmSAG was identified by serum from *E. maxima*-infected chicken; lane 5: purified rEmSAG was identified by serum from coccidia-free chicken.

The rEmSAG protein was expressed in *E. coli* BL21 (DE3) and purified using a His-tag purification column. The purification was verified through SDS-PAGE and immunoblot assays. As shown in Figure 1, the results indicated that compared with before purification (Figure 1, lane 1), the molecular weight of purified rEmSAG is 43 kDa, consistent with theoretical predictions, confirming successful purification (Figure 1, lane 2).

Furthermore, the immunoblot analysis demonstrated that rEmSAG is recognised by a mouse-derived anti-His tag antibody and serum from *E. maxima*-infected chickens (Figure 1, lane 3 and lane 4), but not by serum from coccidia-free chickens (Figure 1, lane 5). This indicates that rEmSAG possesses satisfactory antigenicity and can elicit an immune response in the host against EmSAG.

### Regulatory effects of rEmSAG on the immune function of chicken PBMCs

#### Effect of rEmSAG on the proliferation of chicken PBMCs

Parent generation cells were labeled with a fluorescent dye, and subsequent generations were tracked using flow cytometry through the dye dilution method. The percentage of progeny cells generated by rEmSAG-stimulated chicken PBMCs is presented in Additional file 1. After stimulation with rEmSAG at concentrations of 10, 20, and 40 μg/mL, the percentages of neogenic PBMCs were found to be 54.65 ± 0.8491 (%), 56.86 ± 1.564 (%), and 56.58 ± 0.9765 (%), respectively. These values were all significantly higher than those observed in the pET-32a tag protein (49.31 ± 0.6232) (%) and PBS (50.30 ± 0.3988) (%) control groups (*p* < 0.05). In contrast, the percentage of neogenic PBMCs stimulated with rEmSAG at a concentration of 80 μg/mL was 47.87 ± 0.7139 (%), which was not significantly different from the pET-32a tag protein control group (*p* > 0.05).

**Figure 2:**
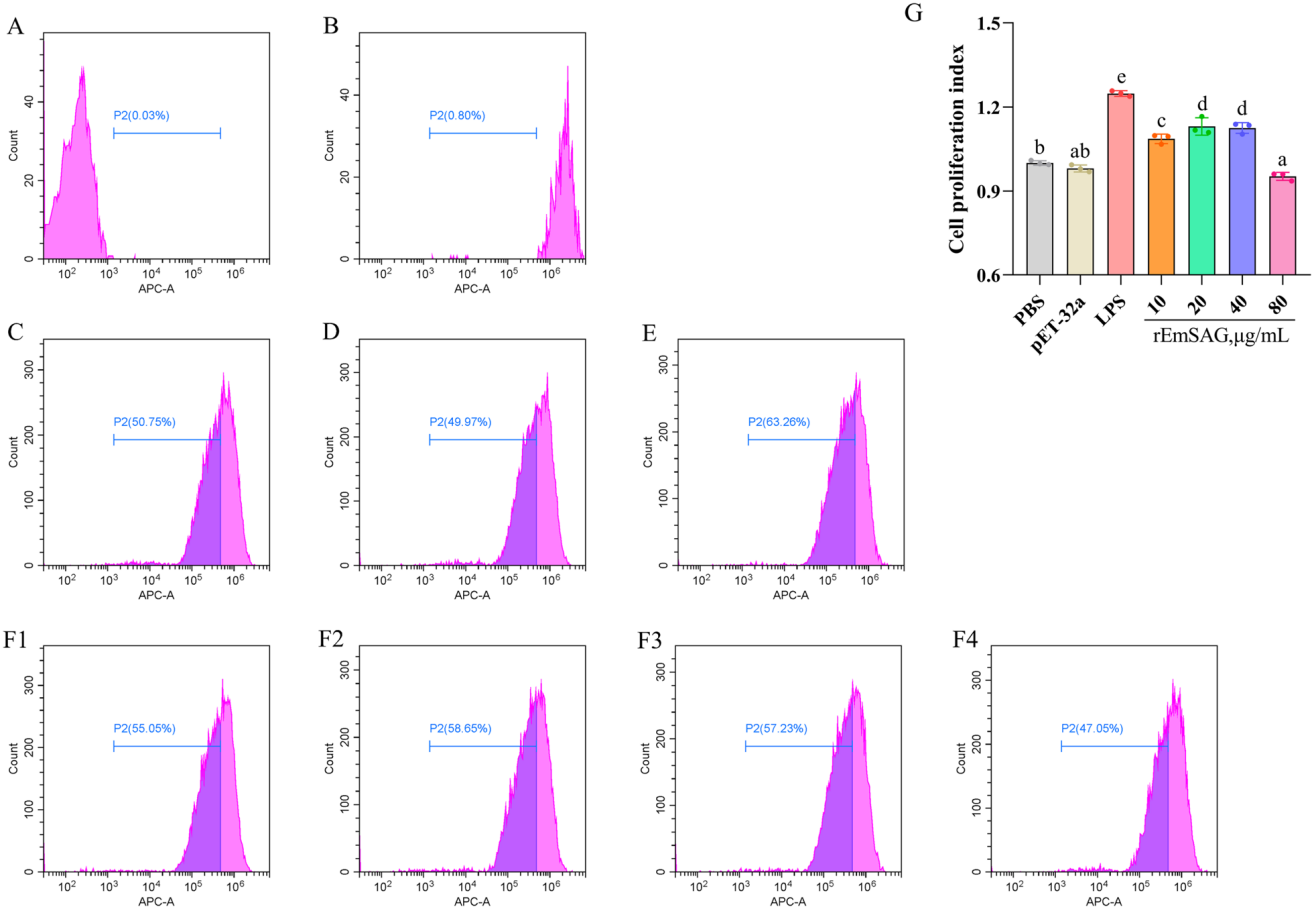
**Detection of cell proliferation of chicken PBMCs by flow cytometry. A**: The auto-fluorescence intensity of the progeny generation cells in chicken PBMCs. **B**: The fluorescence intensity of the parent generation cells in chicken PBMCs. C: The percentage of neogenic chicken PBMCs following stimulation with PBS. **D**: The percentage of neogenic chicken PBMCs following stimulation with pET-32a tag protein. **E**: The percentage of neogenic chicken PBMCs following stimulation with LPS. F1-4: The percentage of neogenic chicken PBMCs following stimulation with 10, 20, 40, and 80 μg/mL rEmSAG, respectively. **G**: The cell proliferation index was used to indicate the effect of rEmSAG on the proliferation in chicken PBMCs.

The flow cytometry histogram data are illustrated in Figures 2A–F. Figures 2A and B display the auto-fluorescence intensity of progeny generation cells and the fluorescence intensity of parent generation cells, respectively. The proliferation percentages of chicken PBMCs after 48 h stimulation are shown in Figures 2C-E (PBS, pET-32a-tagged protein, and LPS) and Figures 2F1–4 (10–80 μg/mL rEmSAG). To quantify the impact of rEmSAG on the proliferative capability of PBMCs, the percentages were calculated and converted into a cell proliferation index, as shown in Figure 2G. Notably, rEmSAG at concentrations of 10, 20, and 40 μg/mL significantly promoted the proliferation of chicken PBMCs (*p* < 0.05). However, the 80 μg/mL rEmSAG concentration did not show a significant difference compared to the PBS and His-tagged pET-32a protein groups (*p* > 0.05).

#### Effects of rEmSAG on T lymphocyte subsets proportion in chicken PBMCs

**Figure 3:**
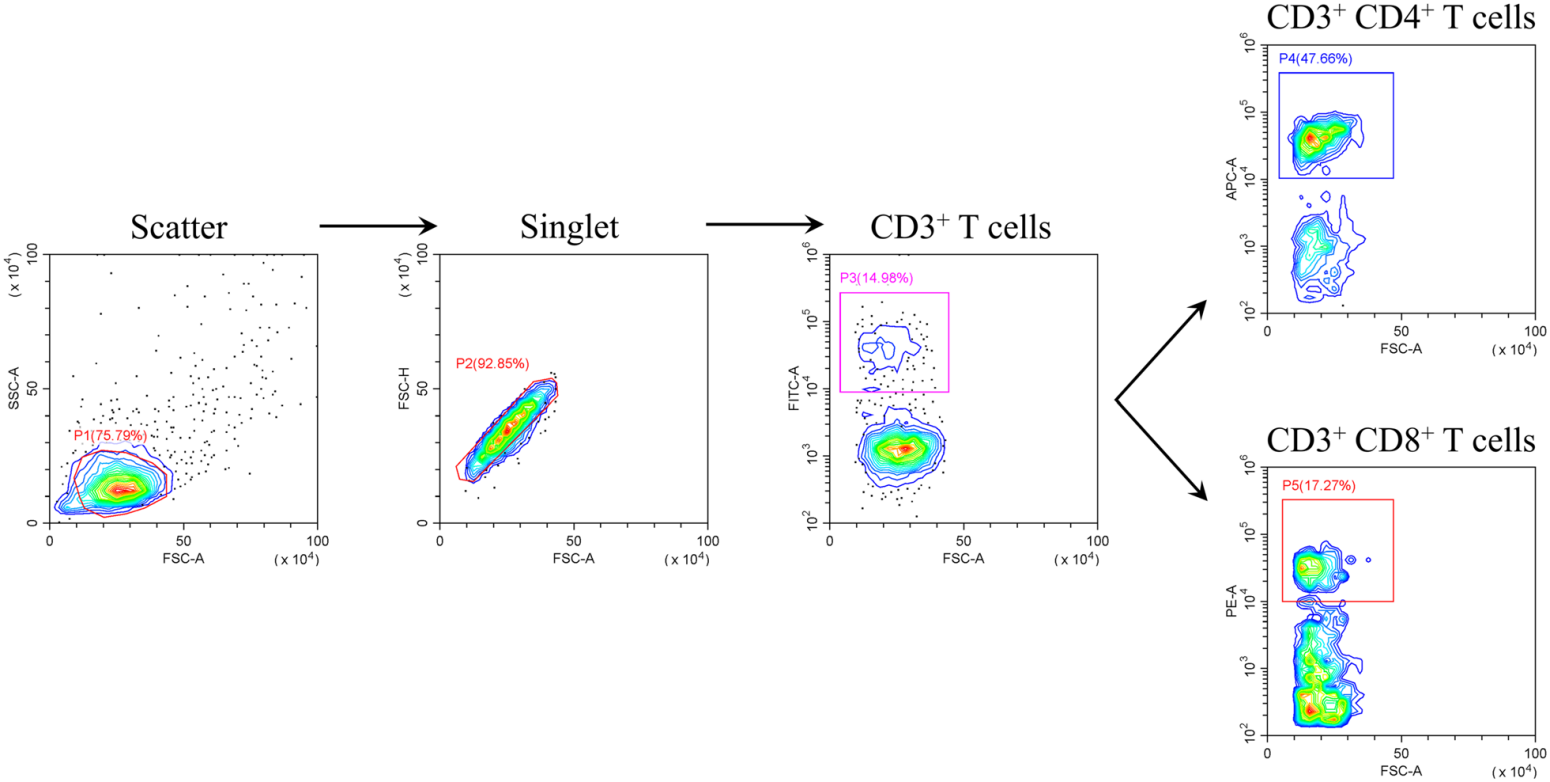
**The flow cytometry serial gating strategy for detection of the proportion of CD4**^**+**^** and CD8**^**+**^** T lymphocytes.** Chicken PBMCs stimulated by different treatment groups were collected and detected by flow cytometry. Cell debris were excluded by gating according to FSC/SSC, and then singlet cells were obtained by gating according to FSC-A/FSC-H. The CD3^+^ T cells were gated by the addition of the isotype control and FITC-labelled specific fluorescent antibodies, and the proportion of CD4^+^ and CD8^+^ T lymphocytes was measured by extracellular labelling of CD4 or CD8 with specific fluorescent antibodies. Results shown are from a representative experiment. FSC: forward scatter, SSC: side scatter, FITC: fluorescein isothiocyanate, APC: allophycocyanin, PE: P-phycoerythrin.

**Figure 4:**
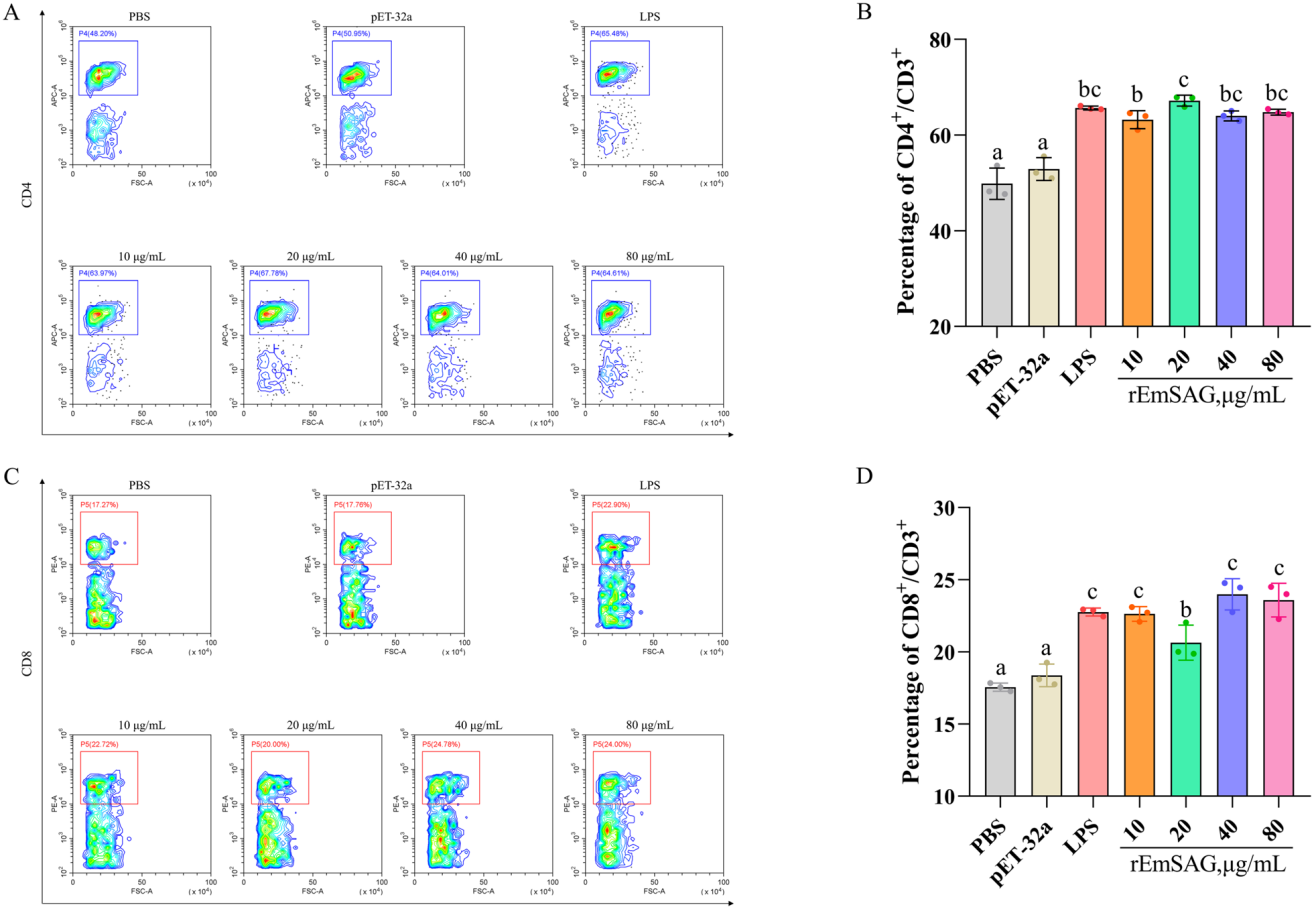
**Investigation of the proportion of CD4**^**+**^**/CD8**^**+**^** T lymphocytes in chicken PBMCs. A**: CD4^+^ T lymphocytes were detected by contour plot of flow cytometry using extracellular surface molecular antibodies (anti-CD3 and anti-CD4). **B**: Proportion of CD4^+^ T cell subset stimulated by different concentrations of rEmSAG. **C**: CD8^+^ T lymphocytes were detected by contour plot of flow cytometry using extracellular surface molecular antibodies (anti-CD3 and anti-CD8). **D**: Proportion of CD8^+^ T cell subset stimulated by different concentrations of rEmSAG.

After incubating rEmSAG-stimulated chicken PBMCs with fluorescent antibodies, we analysed the cell surface markers (CD3, CD4, and CD8) in each treatment group using flow cytometry. The gating strategy used for detecting the proportions of CD4^+^/CD8^+^ T lymphocytes is illustrated in Figure 3. The contour plots in Figure 4A and C display the proportions of CD3^+^CD4^+^ and CD3^+^CD8^+^ T lymphocytes, respectively. As shown in Figure 4B, all concentrations of rEmSAG induced a significant increase in the proportion of CD4^+^ T lymphocytes in chicken PBMCs compared to both the PBS and His-tagged pET-32a protein stimulation groups (*p* < 0.05). Similarly, the proportion of CD8^+^ T lymphocytes was also significantly elevated in response to rEmSAG stimulation (*p* < 0.05), as depicted in Figure 4D.

#### Effects of rEmSAG on nitric oxide release and cytokines transcription in chicken PBMCs

**Figure 5:**
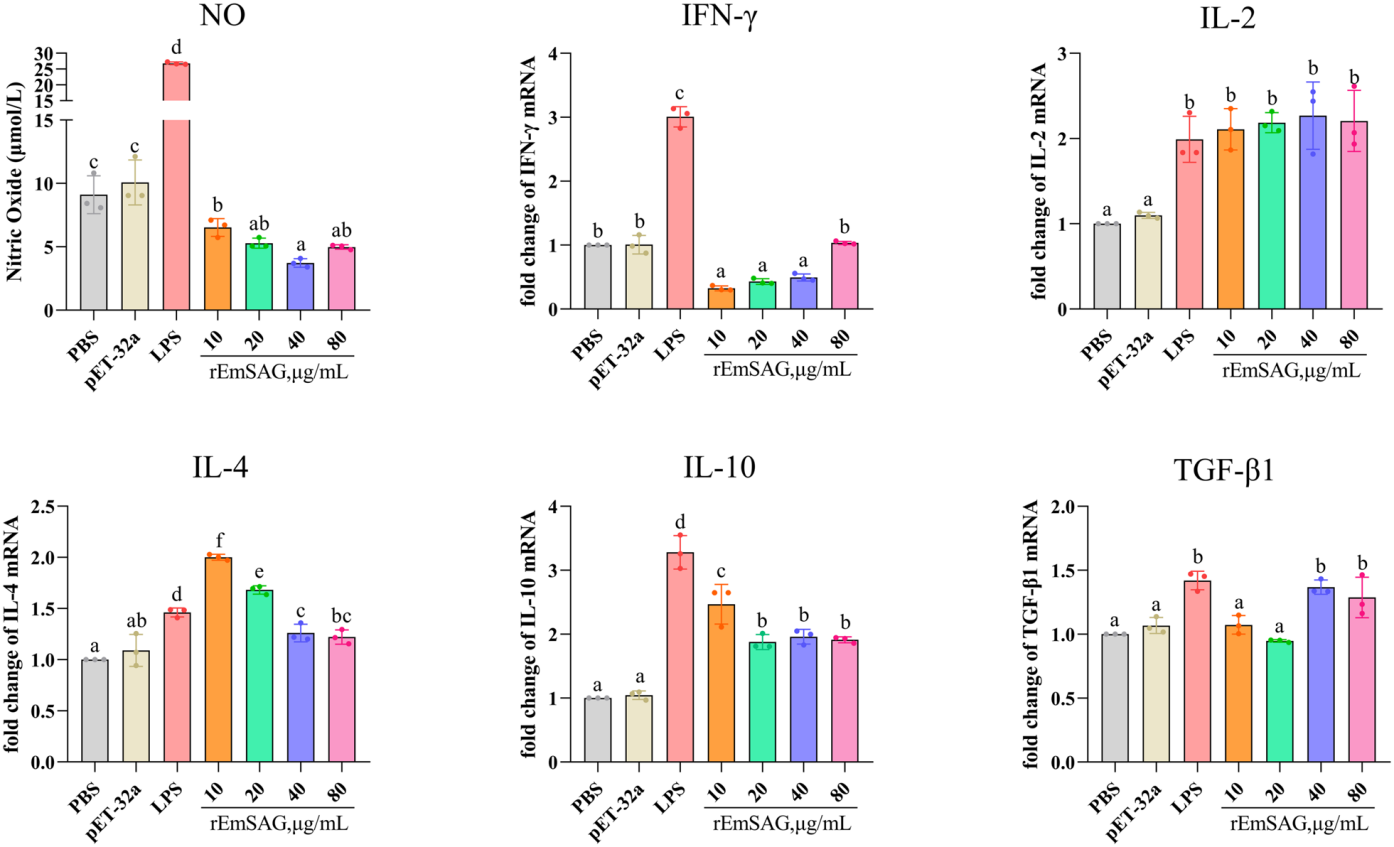
**Detection of total nitric oxide release and cytokines transcription in chicken PBMCs.** The nitrite reduction method was used to determine the concentration of total nitric oxide in chicken PBMCs following incubation with 10, 20, 40, and 80 μg/mL rEmSAG. qPCR was used to detect the transcription of cytokines including IFN-γ, IL-2, IL-4, IL-10, and TGF-β1 after chicken PBMCs were stimulated with 10, 20, 40, and 80 μg/mL rEmSAG.

The effect of rEmSAG on NO release from chicken PBMCs was evaluated using the Griess assay (nitrite quantification). As demonstrated in Figure 5, rEmSAG significantly inhibited NO release from chicken PBMCs at various concentrations (*p* < 0.05). Additionally, the impact of rEmSAG on cytokine transcription in chicken PBMCs was assessed using qPCR. The results shown in Figure 5 indicate that rEmSAG significantly inhibited the transcription of IFN-γ while promoting the transcription of IL-2, IL-4, IL-10, and TGF-β1 in chicken PBMCs (*p* < 0.05).

### Regulatory effects of rEmSAG on the immune function of chicken T cell subsets

#### Assessment of the purity of chicken T cell subsets by flow cytometry

**Figure 6:**
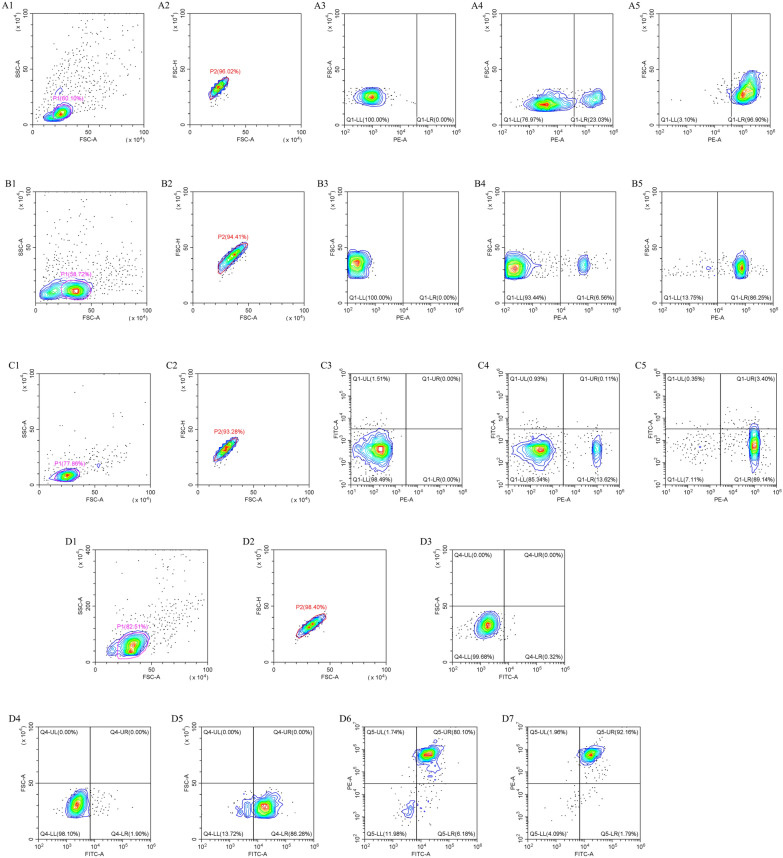
**Detection of cell sorting purity of T cell subsets in chicken by flow cytometry. A**: Magnetic bead sorting of chicken CD8^+^ T cells. **B**: Magnetic bead sorting of chicken CD4^+^ T cells. **C**: Magnetic bead sorting of chicken CD4^+^CD25^−^ T cells. **D**: Magnetic bead sorting of chicken CD4^+^CD25^+^ T cells. 1: Lymphocytes in chicken peripheral blood. 2: Singlet cells. 3: Blank cells incubated without fluorescent antibody. 4: CD8^+^, CD4^+^, CD4^+^CD25^−^, and CD25^+^ cells before cell sorting. 5: CD8^+^, CD4^+^, CD4^+^CD25^−^, and CD25^+^ cells after cell sorting. 6: CD4^+^CD25^+^ T cells before cell sorting. 7: CD4^+^CD25^+^ T cells after cell sorting.

Different T cell subsets were sorted using immunomagnetic bead sorting with indirect labeling, and flow cytometry was employed to assess the purity of the positive cells. The purity levels achieved were as follows: CD8^+^ for 96.90%, CD4^+^ for 86.25%, CD4^+^CD25^−^ for 89.14%, and CD4^+^CD25^+^ Tregs for 92.16% (Figure 6). These purified populations were considered suitable for subsequent co-incubation experiments with rEmSAG.

#### Effects of rEmSAG on cytokines transcription in chicken T cell subsets

**Figure 7:**
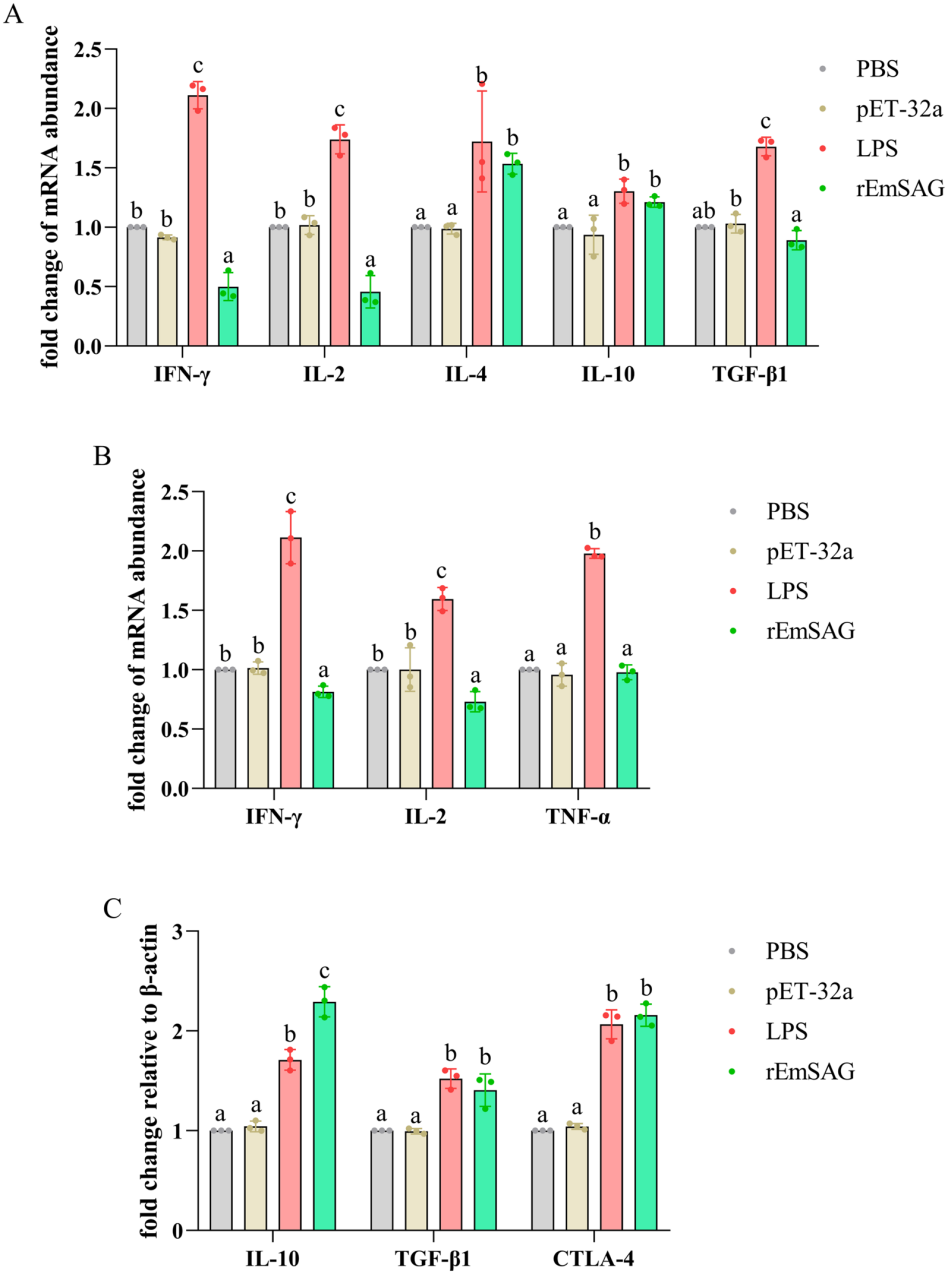
**Effects of rEmSAG on cytokines transcription of T cell subsets in chicken. A**: Relative fold changes in IFN-γ, IL-2, IL-4, IL-10, and TGF-β1 transcription were defined in chicken CD4^+^ T cells following stimulation with 20 μg/mL rEmSAG. **B**: Relative fold changes in IFN-γ, IL-2, and TNF-α transcription were defined in chicken CD8^+^ T cells following stimulation with 20 μg/mL rEmSAG. **C**: Relative fold changes in IL-10, TGF-β1, and CTLA-4 transcription were defined in chicken CD4^+^CD25^+^ T cells following stimulation with 20 μg/mL rEmSAG.

In this study, we investigated chicken T cell subsets, including CD8^+^, CD4^+^, and CD4^+^CD25^+^ Tregs, by stimulating them with rEmSAG for qPCR assays. The results demonstrated that stimulation with rEmSAG significantly inhibited the transcription of IFN-γ and IL-2 while promoting the transcription of IL-4 and IL-10 in CD4^+^ T cells (*p* < 0.05; Figure 7A).

Similarly, in CD8^+^ T cells, rEmSAG significantly reduced the transcription levels of IFN-γ and IL-2 (*p* < 0.05; Figure 7B). However, there was no statistically significant effect observed on the transcription level of TNF-α (*p* > 0.05; Figure 7B). Additionally, rEmSAG significantly enhanced the expression of hallmark cytokines (IL-10 and TGF-β1) and the surface marker (CTLA-4) in chicken Tregs (*p* < 0.05; Figure 7C).

#### Effects of rEmSAG on cytokines transcription of chicken CD4^+^CD25^−^ T cells following CD25^+^ cell depletion

**Figure 8:**
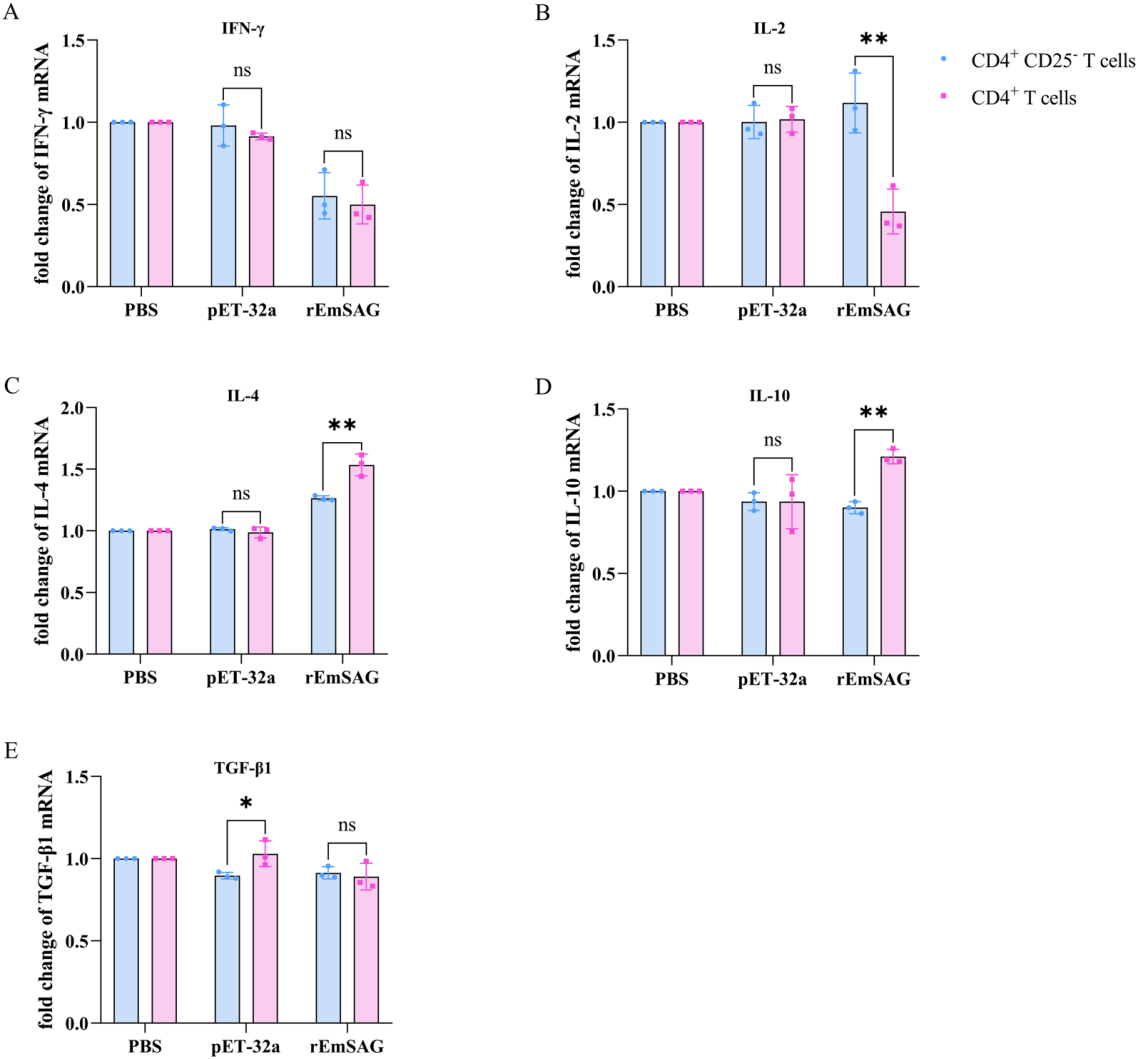
**Effects of rEmSAG on cytokines transcription of chicken CD4**^**+**^**CD25**^**−**^** T cells following CD25**^**+**^** cell depletion.** qPCR was used to detect the transcription of cytokines including IFN-γ, IL-2, IL-4, IL-10, and TGF-β1 in chicken CD4^+^CD25^−^ T cells stimulated with 20 μg/mL rEmSAG following the depletion of CD25^+^ cells from CD4^+^ T cells.

To investigate the regulatory role of CD25⁺ T cells, we stimulated both CD4⁺CD25⁻ T cells (which were depleted of the CD25⁺ population) and conventional CD4⁺ T cells with 20 μg/mL rEmSAG for a comparative analysis of the transcriptional analysis of Th1, Th2, and Treg-related cytokines. The results showed that rEmSAG significantly increased the transcription level of IL-2 in chicken CD4^+^CD25^−^ T cells compared to chicken CD4^+^ T cells (*p* < 0.05; Figure 8B). Conversely, rEmSAG significantly decreased the transcription levels of IL-4 (*p* < 0.05; Figure 8C) and IL-10 (*p* < 0.05; Figure 8D). However, there were no significant differences in the transcription levels of IFN-γ (*p* > 0.05; Figure 8A) and TGF-β1 (*p* > 0.05; Figure 8E). These findings suggest that the depletion of CD25^+^ T cells can significantly reverse the inhibitory effect of rEmSAG on IL-2 transcription in chicken CD4^+^CD25^−^ T cells, while also reducing its promotion of IL-4 and IL-10.

## Discussion

Chicken coccidiosis is a major disease that poses a significant threat to the global poultry industry. The prevention and control of this disease is increasingly challenging due to rising drug resistance and concerns over drug residues [4, 5]. Additionally, similar to other parasitic protozoa, *Eimeria* spp. employ various strategies to evade the host immune system. One effective strategy is the secretion of immunomodulatory molecules that inhibit the production of pro-inflammatory cytokines while regulating the activation, proliferation, and differentiation of T and B lymphocytes.

Our prior research identified four molecules that inhibit IFN-γ, one of which is EmSAG (unpublished data). Given the crucial role of the IFN-γ-mediated Th1 immune response in anticoccidial immunity [1], the suppression of IFN-γ by pathogen-derived factors may be a key mechanism for *Eimeria* spp. to evade the immune response. Moreover, IL-10 produced by Tregs is recognised as another contributor to *Eimeria’s* immune evasion [20].

This study investigates the dual immunoregulatory function of EmSAG in chicken PBMCs and T cell subsets, specifically its role in suppressing IFN-γ production and inducing IL-10 and TGF-β1. Notably, the depletion of CD25⁺ cells partially reduced the EmSAG-mediated inhibition of IFN-γ, suggesting that CD25⁺ cells are involved in this regulatory network. Mechanistically, EmSAG appears to facilitate immune evasion by coordinating the suppression of the Th1 response and the activation of Tregs. Our research provides important insights into the immune evasion mechanisms used by *Eimeria*.

The proliferation, differentiation, and functional collaboration of immune cells are crucial for host defense against *Eimeria* infections. In chickens, PBMCs (mainly lymphocytes and monocytes) and CD4^+^/CD8^+^ T cells can be activated to respond to various *Eimeria* infections [1, 24, 25]. To investigate the immunomodulatory effects of EmSAG, we assessed its impact on PBMC proliferation and the differentiation of CD4^+^/CD8^+^ T cells using flow cytometry. Our results demonstrate that rEmSAG, at concentrations ranging from 10–40 μg/mL (with the peak effect observed at 20 μg/mL), effectively stimulated PBMC proliferation. Additionally, rEmSAG enhanced the surface expression of CD4 and CD8 markers and promoted the differentiation and maturation of both T cell subsets.

These findings regarding the proliferation of chicken immune cells induced by EmSAG align with established mechanisms of anticoccidial immunity. For instance, CD4^+^ intraepithelial lymphocytes (IELs) and lamina propria lymphocytes (LPLs) proliferate in the duodenum and cecum during infections caused by *E. acervulina* and *E. tenella* [26–28]. Moreover, CD8^+^ T cells assist in the transport and control of sporozoite spread, migrating to the cecal tonsils in infected chickens [27, 29, 30]. Overall, these results suggest that EmSAG may activate the immune response, establish immune memory, or enhance immune protection, ultimately helping chickens resist coccidiosis.

Studies conducted both in vitro and in animal models have shown that nitric oxide, which is produced in response to cytokine stimulation, plays a vital role in mediating immune protection against various protozoa infections [31–33]. In avian coccidiosis models, Allen observed increased plasma levels of NO_2_^−^ and NO_3_^−^ at 5 days after *E. tenella* infection [34]. Furthermore, chicken spleen macrophages stimulated by *E. tenella* sporozoites exhibited heightened NO synthesis, particularly at parasitic sites [35].

In experiments with *E. tenella*-infected primary chicken kidney cells (PCKs), the addition of exogenous NO from sodium nitroferricyanide (III) dihydrate (SNP) led to increased excretion of free sporozoites within just 30 min, suggesting that NO stimulates rapid sporozoite release [36]. On the other hand, impaired NO synthesis can increase the host’s susceptibility to parasitic protozoa [37–41].

In this study, we quantified NO metabolites (NO_2_^−^ and NO_3_^−^) in the supernatant of chicken PBMCs stimulated with *E. maxima* recombinant SAG protein (rEmSAG) using the Griess assay. The results showed that rEmSAG significantly inhibited NO secretion. This finding is supported by research conducted by Chow et al. on seven *E. tenella* SAG family members, which revealed that macrophages produced low levels of NO when stimulated by these SAG family members, including rEtSAG2 and rEtSAG3 [42]. This aligns with the NO-inhibitory phenotype we observed in our study.

Th1 immune response plays a crucial role in resisting infections caused by parasitic protozoa. However, these parasites have developed mechanisms to weaken the Th1 immune response by inhibiting the secretion of IFN-γ or IL-12, which allows them to evade the immune system and sustain persistent infections [7–11]. In particular, the early activation of IFN-γ is essential for combating *E. maxima* infections [43]. Our earlier research showed that *Eimeria* spp. can disrupt the Th1 immune response by suppressing IFN-γ levels. When chickens were injected with a recombinant plasmid and protein encoding the SAG gene of *E. maxima*, serum IFN-γ levels decreased, while IL-4, IL-10, and TGF levels increased at 6 dpi (unpublished data). Mechanistically, in vitro experiments demonstrated that rEmSAG inhibits IL-12p40 expression in HD11 cells and BMDCs by regulating phosphorylated ERK1/2, which subsequently reduces IFN-γ secretion [14]. This study builds on those findings by systematically assessing the immunomodulatory effects of EmSAG in chicken PBMCs and T cell subsets. The qPCR assay showed significant inhibition of IFN-γ transcription in chicken PBMCs, as well as in CD4^+^ and CD8^+^ T cells, following EmSAG stimulation, highlighting its effectiveness as an IFN-γ inhibitory molecule.

Notably, as the concentration of rEmSAG increased, its inhibitory effect on IFN-γ was gradually diminished. At a concentration of 80 μg/mL, the transcription level of IFN-γ in chicken PBMCs showed no significant reduction. This lack of effect is likely due to the functional concentration exceeding the optimal range (10–40 μg/mL). We maintain that the inhibitory effect of rEmSAG on IFN-γ production by chicken PBMCs requires maintaining an appropriate concentration. If this concentration surpasses a specific threshold, the protein's impact on IFN-γ secretion may change direction.

The functional concentration range of EmSAG is supported by complementary cell proliferation assays. Data presented in Figure 2G shows that rEmSAG at concentrations of 10, 20, and 40 μg/mL significantly promoted chicken PBMC proliferation, whereas 80 μg/mL did not exhibit any proliferative effect tended to be inhibitory. This could explain why the results deviate from the initial working hypothesis. However, further studies are needed to explore the more complex mechanisms that may be involved.

Another important factor to consider is the potential deviations in the relative quantification method of qPCR, which occur due to variations in the selection of standard housekeeping genes. For example, some anti-parasitic drugs inhibit the housekeeping gene *β-tubulin*. This could lead to errors in detecting the relative fold changes of gene expression in drug-resistant strains if β-tubulin is used as a housekeeping gene.

In the present study, we used the qPCR relative quantification method to measure the relative fold changes of cytokine mRNA levels in PBMCs and T cell subsets of healthy chickens. It is important to note that the chickens used in this experiment were not administered any anticoccidial drugs. Therefore, we are inclined to move away from the initial working hypothesis due to the high concentration (80 μg/mL) of rEmSAG.

Research on the immune evasion strategies of parasitic protozoan emphasises the role of host CD4^+^CD25^+^FoxP3^+^ regulatory T cells (Tregs; in chickens, defined as CD4^+^CD25^+^ T cells due to the lack of FoxP3 gene). Tregs regulate the proliferation and activation of immune cells through various mechanisms that include the release of regulatory cytokines (IL-10, TGF-β, IL-35), as well as the action of granzyme B, perforin-1, and specific surface markers (CTLA-4, PD-1, LAG-3) [44]. These molecules are notably upregulated in Tregs during infections with parasitic protozoa and act as key players in immunosuppression [16, 45–49].

In the case of avian coccidiosis, infection with *Eimeria* triggers the expansion of Tregs, which is associated with increased expression of IL-10, TGF-β, and CTLA-4. For instance, Selvaraj et al. demonstrated that coccidia infection induces Treg differentiation [18]; Han et al. found an accumulation of intestinal Tregs during *E. tenella* infection, with heightened IL-10 production contributing to immunosuppression [50]. Additionally, Yu et al. reported that Tregs-secreted IL-10, TGF-β, and CTLA-4 in the cecum peaked at 6 days post-*E. tenella* infection, while splenic IL-10 peaked at 6 dpi and TGF-β/CTLA-4 peaked at 10 dpi [19].

These findings closely align with our experimental results. Specifically, rEmSAG stimulation led to a significant increase in the expression of IL-10 and TGF-β1 in PBMCs, IL-10 in CD4^+^ T cells, and IL-10, TGF-β1, and CTLA-4 in Tregs. This suggests that the SAG of *E. maxima* may play a role in the disease process of chicken coccidiosis. One possible hypothesis is that coccidia have developed mechanisms to stimulate Tregs to produce IL-10, thereby inhibiting the IFN-γ-mediated Th1 immune response and enhancing parasite survival. However, current evidence does not directly support the notion that EmSAG-induced inhibition of IFN-γ is mediated through IL-10 induction.

We temporarily hypothesised that rEmSAG may promote Treg activation, leading to the secretion of IL-10 or TGF-β, which in turn inhibits the expression of IFN-γ and IL-2. To validate this mechanism, we will conduct CD25^+^ cell depletion studies to explore the correlation between the inhibition of IFN-γ/IL-2 and the activation of Tregs.

Given the role of Tregs in promoting disease progression, researchers have started to explore strategies to disrupt Tregs-mediated immunosuppression through approaches such as cellular depletion, cytokine neutralisation, and the inhibition of surface molecules. One strategy involves depleting host Tregs to reduce susceptibility to infections such as *Plasmodium*, *Leishmania*, and *Trypanosoma* [51–53].

Therapeutic neutralisation of cytokines produced by Tregs, including IL-10 [54, 55], TGF-β [16], IL-35 [16] and checkpoint inhibitors-CTLA-4 [56], PD-1 [57], LAG-3 [57, 58] via monoclonal antibodies (mAbs), has the potential to reduce host damage and enhance parasite clearance. In avian coccidia models, for instance, the intraperitoneal injection of anti-CD25 mAbs leads to an approximate 80% reduction in intestinal Tregs, which subsequently elevates mRNA levels of IFN-γ and IL-2, and promotes the proliferation of CD4^+^CD25^−^ T cells [59, 60].

Similarly, the use of anti-IL-10/IL-10R antibodies has been shown to increase IFN-γ levels, reduce intestinal lesions, and decrease immunosuppression induced by IL-10 [61–64]. In this study, depleting CD25^+^ cells significantly reversed the inhibition of IL-2 and lessened the promotion of IL-4/IL-10 caused by rEmSAG, while higher levels of IFN-γ were observed. These results suggest that rEmSAG predominantly inhibits IFN-γ expression directly, with some involvement in the induction of Tregs-secreted IL-10, which also inhibits IFN-γ. This conclusion is further supported by the observation that the transcription level of IFN-γ did not show a significant difference after CD25^+^ cell depletion.

However, the mechanism of rEmSAG-mediated IL-2 inhibition operates via dual pathways: a Tregs-dependent inhibition via IL-10 production and a direct inhibition of IL-2 expression in CD4^+^CD25^−^ T cells or CD8^+^ T cells. The reversal of IL-2 inhibition following CD25^+^ cell depletion could confirm the existence of this dual regulatory pathway.

In conclusion, this study clarified the effects of the *E. maxima* IFN-γ inhibitory molecule, EmSAG, on the immune functions of chicken PBMCs and T cell subsets, with a focus on adaptive immunity. Stimulation with rEmSAG suppressed NO release and IFN-γ levels while upregulating IL-4, IL-10, and TGF-β1 in PBMCs. Additionally, it inhibited IFN-γ in CD4^+^/CD8^+^ T cells and promoted IL-10 level in CD4^+^/CD4^+^CD25^+^ subsets. Depleting CD25^+^ cells reversed the suppression of IL-2 and diminished the rEmSAG-induced promotion of IL-4/IL-10, while also enhancing PBMC proliferation and the differentiation of CD4^+^/CD8^+^ T cells. These findings provide critical insights into the immune evasion mechanisms utilised by chicken coccidia through the Th1 immune response and the function of Treg cells, paving the way for potential therapeutic strategies against *E. maxima* infections.

## Supplementary Information

**Additional file 1. The percentage of progeny generation cells produced by rEmSAG stimulated chicken PBMCs**.

## Data Availability

All data generated or analysed in this research are included in this paper and its additional information files.
