# Supplementary material for: Immunomodulatory effects of Eimeria maxima surface antigen (EmSAG) as an IFN-γ inhibitory molecule on peripheral blood mononuclear cells (PBMCs) and T cell subsets in chickens
Source: Vet Res. 2025 May 19;56:103. doi: 10.1186/s13567-025-01535-7 (PMC12090499; doi:10.1186/s13567-025-01535-7)
Supplement: Supplementary file 1 — Additional file 1. The percentage of progeny generation cells produced by rEmSAG stimulated chicken PBMCs. [file 13567_2025_1535_MOESM1_ESM.docx]

**Additional file 1 The percentage of progeny generation cells produced by rEmSAG stimulated chicken PBMCs.**

| **Groups** | | **Percentage of progeny generation cells (%)** | | | |
| --- | --- | --- | --- | --- | --- |
|  |  | sample 1 | sample 2 | sample 3 | Mean value |
| PBS | | 50.75 | 50.00 | 50.14 | 50.30 ± 0.3988^b^ |
| pET-32a | | 49.24 | 49.97 | 48.73 | 49.31 ± 0.6232^ab^ |
| LPS | | 62.25 | 63.26 | 62.80 | 62.77 ± 0.5057^e^ |
| rEmSAG | 10 μg/mL | 53.66 | 55.05 | 55.20 | 54.64 ± 0.8491^c^ |
|  | 20 μg/mL | 56.17 | 55.76 | 58.65 | 56.86 ± 1.564^d^ |
|  | 40 μg/mL | 57.06 | 55.46 | 57.23 | 56.58 ± 0.9765^d^ |
|  | 80 μg/mL | 48.25 | 48.32 | 47.05 | 47.87 ± 0.7139^a^ |

In the present experiment, the same superscript letter (a-e) indicates a nonsignificant difference between the two groups (*p* > 0.05), while the different superscript letters a-e indicate a significant difference between the two groups (*p <* 0.05).
